# The Development of Ru(II)-Based Photoactivated Chemotherapy Agents

**DOI:** 10.3390/molecules26185679

**Published:** 2021-09-18

**Authors:** Yongjie Chen, Lijuan Bai, Pu Zhang, Hua Zhao, Qianxiong Zhou

**Affiliations:** 1 Research Center for Pharmacodynamic Evaluation Engineering Technology of Chongqing, College of Pharmacy, Chongqing Medical University, Chongqing 400016, China; bailijua88@126.com (L.B.); zhangpu51@hotmail.com (P.Z.); 100923@cqmu.edu.cn (H.Z.); 2 Key Laboratory of Photochemical Conversion and Optoelectronic Materials, Technical Institute of Physics and Chemistry, Chinese Academy of Sciences, Beijing 100190, China

**Keywords:** photoactivated chemotherapy (PACT), Ru(II) complexes, photoinduced ligand dissociation

## Abstract

Photoactivated chemotherapy (PACT) is a novel cancer treatment method that has drawn increasing attention due to its high selectivity and low side effects by spatio-temporal control of irradiation. Compared with photodynamic therapy (PDT), oxygen-independent PACT is more suitable for treating hypoxic tumors. By finely tuning ligand structures and coordination configurations, many Ru(II) complexes can undergo photoinduced ligand dissociation, and the resulting Ru(II) aqua species and/or free ligands may have anticancer activity, showing their potential as PACT agents. In this mini-review, we summarized the progress in Ru(II)-based PACT agents, as well as challenges that researchers in this field still face.

## 1. Introduction

**Figure 1:**
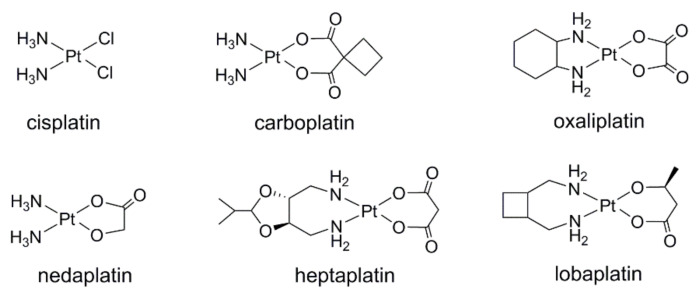
Clinically approved Pt (II) anticancer drugs.

The research of new chemotherapeutic drugs for cancer treatment has been the focus in medicine and related fields for many years. Among them, the development of transition-metal-based agents has always been one of the hotspots [1,2,3,4,5,6,7], which obviously benefits from the long-term and wide clinical application of cisplatin [*cis*-Pt (NH_3_)_2_Cl_2_]. The unexpected discovery by Rosenberg and his co-workers revealed the antitumor activity of cisplatin in the 1960s [8,9]; subsequently, cisplatin and its analogs (Figure 1) have been successfully used in the clinical treatment of many cancers [10]. Although cisplatin and its derivatives are efficacious against the vast majority of cancers, their selectivity to tumor tissues and normal tissues is poor [11]. This leads to the production of non-cancer cell toxicity; thereby, severe adverse effects are caused, including hair loss, peripheral neuropathy, and myelotoxicity in patients [12,13,14].

Many strategies have been used for solving the above problems, for example, using targeting drug delivery systems and developing reduction responsive Pt(IV) prodrugs [15,16]. In addition, many photoactivated Pt(IV) agents have been reported, which are nontoxic in the dark but can release cytotoxic Pt(II) species upon light irradiation [17,18]. These kinds of prodrugs are commonly known as photoactivated chemotherapy (PACT) agents, which can trigger and limit the drug activity within the tumor tissues by spatio-temporal control of irradiation, thus achieving fewer side effects [19,20,21]. Compared with traditional photodynamic therapy (PDT) [22,23], another phototherapy that is generally oxygen-dependent, PACT offers an oxygen-independent mechanism, and is therefore more suitable for hypoxic tumors, where the concentration of oxygen is remarkably low [24,25].

**Figure 2:**
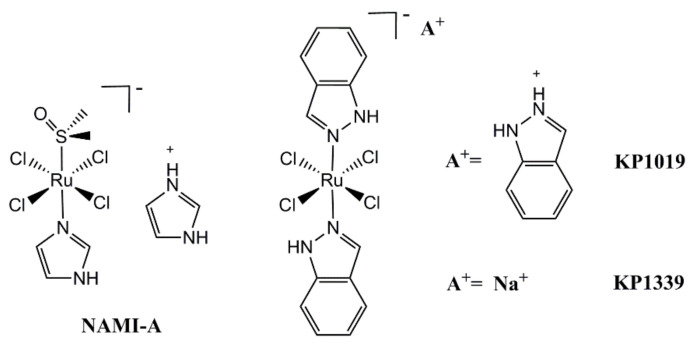
Three ruthenium(III) complexes in clinical trials.

Inspired by the success of Pt complexes as antitumor drugs, other transition metal complexes, especially Ruthenium (Ru) complexes, have drawn great attention owing to their potential anticancer properties and selective cytotoxic activity [26]. Investigating new Ru-based complexes as anticancer drugs is an important trend in modern medicinal inorganic chemistry [27,28,29,30]. Up till now, several Ru(III) complexes as chemotherapeutic agents have already entered clinical trials, such as NAMI-A, KP1019, and KP1339 (Figure 2) [31,32,33,34,35]. As the first approved Ru complex to reach clinical investigations, NAMI-A showed success in phase I clinical studies but showed only limited efficacy in the phase II stage, which resulted in the failure of the clinical investigations [27,36]. Afterward, another Ru therapeutic KP1019 showed solubility limited in phase I, but in its place, a more soluble sodium salt, KP1339, is currently undergoing clinical trials [27]. Different from focusing on the chemotherapeutic activity of Ru(III) complexes, more attentions has been paid to the photoactivation of Ru(II) complexes. TLD1433, the first Ru(II)-based photosensitizer for PDT, has already entered human clinical trials [37]. Moreover, studies have also indicated that Ru(II) complexes may possess potential as PACT agents [38,39,40,41]. Through proper structure design, they can undergo photoinduced ligand dissociation; then, the resulting Ru(II) aqua species can covalently bind to DNA in a manner similar to cisplatin. Compared with Pt(IV) complexes, Ru(II)-based PACT agents have some promising advantages. They possess diverse and easy-modified structures, rich photophysical and photochemical properties, and furthermore, the octahedral structures different from that of cisplatin may endow them with good activity against cisplatin-resistant cancer cells [42,43,44].

**Figure 3:**
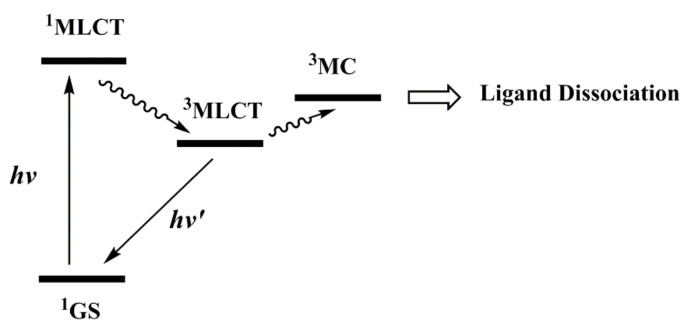
Jablonski diagram of Ru(II) complexes with photolabile ligands.

The lowest energy absorption band of Ru(II) complexes usually comes from ^1^MLCT (Metal-to-Ligand Charge Transfer) transitions. When irradiated by appropriate light, Ru(II) complexes first achieve the ^1^MLCT state and then reach the ^3^MLCT state through ultra-fast intersystem crossing (Figure 3) [19]. The ^3^MLCT state of Ru(II) complexes can return to the ground state through non-radiative inactivation or luminescence pathways [45,46] or can interact with other molecules such as O_2_ to generate singlet oxygen (^1^O_2_), showing potential as photodynamic agents [47,48,49]. This paper focuses on another photochemical process: the ^3^MLCT excited state of Ru(II) complexes may populate the ^3^MC state (metal-centered state or ligand-field state) by thermal activation. The ^3^MC state has M-L (σ*) character, which may lead to ligand dissociation and generate Ru(II) aqua species with DNA-binding ability, showing potential in photoactivated chemotherapy. This mini-review aims to present the latest progress in photoinduced ligand dissociation related Ru(II)-based PACT agents for cancer treatment. By reading this article, we hope to not only let our peers know about the all-around development of Ru(II)-based PACT drugs but also inspire more researchers to enter this interesting field.

## 2. Ligand Photodissociation of Ru(II) Polypyridyl Complexes

Theoretically, the activity of Ru(II)-based PACT agents is closely related to their photoinduced ligand dissociation efficiency. Therefore, the research on ligand dissociation efficiency is the focus of developing potential Ru(II)-based PACT drugs.

### 2.1. Photodissociation of Monodentate Ligands

**Figure 4:**
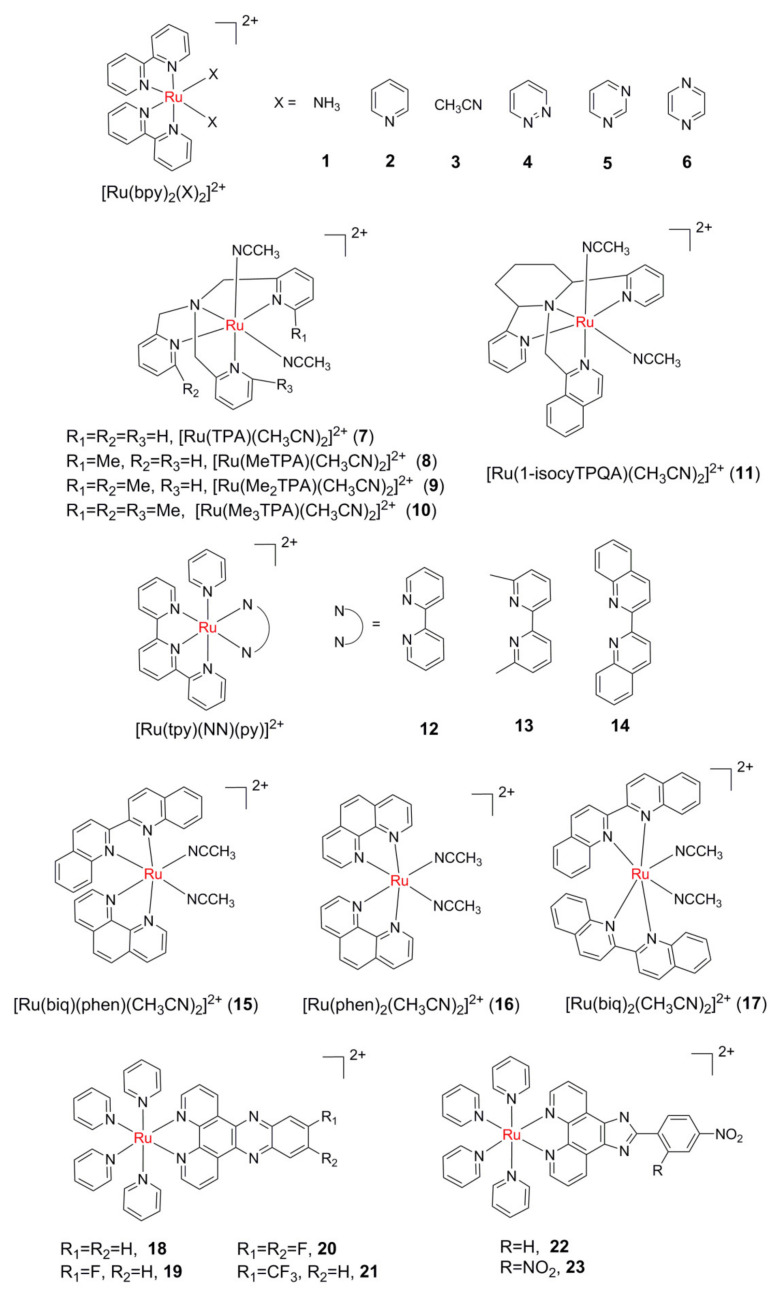
Ru(II) polypyridyl complexes with photolabile monodentate ligands.

The development of photoinduced ligand dissociation and consequently DNA binding of Ru(II) complexes began with a report by Turro and colleagues in 2004 [50]. The photolysis of *cis*-[Ru(bpy)_2_(NH_3_)_2_]^2+^ (bpy = 2,2′-bipyridine, complex **1** in Figure 4) in water was investigated. Irradiation of **1** resulted in the sequential loss of the NH_3_ ligands and the formation of the corresponding bis-aqua complex *cis*-[Ru(bpy)_2_(H_2_O)_2_]^2+^. The photodissociation of ligands was dependent on the excitation wavelength. Under 350 nm and 400 nm light, the ligand exchange quantum yields were 0.024 and 0.018, respectively. Upon irradiation, **1** can form covalent binding with 9-methylguanine, 9-ethylguanine single-stranded and double-stranded DNA.

The photodissociation efficiency of the monodentate ligand is closely related to its coordination ability. Compared with **1**, the photoinduced ligand exchange quantum yield for *cis*-[Ru(bpy)_2_(py)_2_]^2+^(py = pyridine, **2**) between pyridine and Cl^-^ was only 0.0059 [51], while the quantum yield of *cis*-[Ru(bpy)_2_(H_2_O)_2_]^2+^ formed by illumination of *cis*-[Ru(bpy)_2_(CH_3_CN)_2_]^2+^(**3**) in water was as high as 0.38 (350 nm) and 0.22 (450 nm) [52]. The photoinduced ligand exchange reaction of **1** and **3** in an aqueous solution was studied using the density functional theory (DFT) by Kayanuma and co-workers [53]. The ^3^MC structure was lower in energy than the ^3^MLCT structure for **3**; in contrast, the ^3^MLCT structure was lower in energy than the ^3^MC structure for **1**. Such a difference would correlate with the higher photoinduced ligand dissociation quantum yield of **3** compared to **1**.

Glazer et al., reported three Ru(II) polypyridyl complexes **4**–**6** [54] with isomeric diazine ligands, and their photochemical properties were compared to **2**. It was found that unlike **2**, which photodissociated only one monodentate ligand in water, **4**–**6** released both monodentate ligands, with the quantum yields of the second phase varying from the ligand pKa and medium pH. These results suggest that the photochemistry of these complexes can be tuned by simple electronic effects.

The photodissociation efficiency of the monodentate ligand is also related to the distortion degree of octahedral Ru(II) complexes. Thus, the steric bulk is introduced to distort the octahedral field to lower the energy of the ^3^MC state and reduce the energy gap between the ^3^MC state and ^3^MLCT state, which will finally result in greater yields of ligand photodissociation. The photochemical behavior of a series of complexes [Ru(TPA)(CH_3_CN)_2_] (TPA = tris[(pyridin-2-yl)methyl]amine, **7**–**10**) reflects the steric effect very well [55]. The quantum yield of photosubstitution in **7** was measured as 0.009 (400 nm) in water, while that of **8** was 0.011 under the same conditions. Due to the large steric crowding of **9** and **10**, the CH_3_CN ligand can be released even in dark conditions. Lately, complex [Ru(1-isocyTPQA)(CH_3_CN)_2_]^2+^ (1-isocyTPQA = 1-(((2R,6S)-2,6-bis(pyridin-2-yl)piperidin-1-yl)-methyl)isoquinoline, **11**) was designed and synthesized by Kodanko and co-workers, which exhibited unique photochemical properties distinct from **7**–**10** [56]. It was found that the greatly enhanced quantum efficiency (Φ_400_ = 0.033 in water) of photodissociation in **11** can be explained by a trans-type effect in the ^3^MLCT state. DFT calculations and ultrafast transient spectroscopy revealed that a highly mixed ^3^MLCT/^3^ππ* excited state instead of ^3^MC is the lowest-energy triplet state of **11**. This research validated that orbital mixing and steric effects can have a strong influence on photoinduced ligand exchange.

The steric bulk effect is also found in complexes **12–14** [57]. The pyridine dissociation quantum yield upon 500 nm irradiation in CH_3_CN was 0.16 and 0.033 for **13** and **14**, respectively. While under the same conditions, ligand exchange was not observed in **12**, which lacks steric strain. The ligand photodissociation behavior of *cis*-[Ru(biq)(phen)(CH_3_CN)_2_]^2+^(biq = 2,2′-biquino-line, phen = 1,10-phenathroline, **15**) is very unique [58]. Upon irradiation with low energy light (≥550 nm), only one CH_3_CN ligand can exchange with the solvent molecule, but both ligands exchanged with the solvent molecule upon irradiation with high energy light(≥420 nm). In contrast, two CH_3_CN ligands of **16** and **17** were able to exchange with solvent molecules under both light conditions. The theoretical calculations gave a certain explanation for these results. The photodissociation of the ligand of **15** may occur through a so-called “dissociative” mechanism, in which the ligand leaves first to produce five-coordination intermediates. DFT calculations showed that the coordination vacancy of the five-coordination intermediate with the lowest energy forms a *trans* configuration with phen, which means that the CH_3_CN ligand trans to phen leaves preferentially.

Recently, Glazer’s group investigated the interplay of steric and electronic features that impact the photodissociation efficiency of monodentate ligand for Ru(II) complexes [59]. The experimental results showed that rationally varying the ligand components and structures of the metal complex could result in a better photodissociation efficiency.

Additionally, to explore the role of modification on the retaining ligands in the anticancer activity of Ru(II) PACT agents, Zhou et at. reported complexes **18**–**21**, which can photorelease one pyridine ligand and covalently bind to DNA [60]. The study found that compared with **18**, the fluorinated complexes **19**–**21** exhibited enhanced phototoxicity, yet diminished dark cytotoxicity, more favorable for PACT applications. Lately, by introducing the strong electron-withdrawing –NO_2_ groups, two mitochondria-localized complexes **22**–**23** were designed and synthesized [61]. These two complexes can photo-catalyze NADH (β-nicotinamide adenine dinucleotide) depletion and photoinduce ligand dissociation to damage the mitochondrial DNA simultaneously, thus display good activity towards cisplatin-resistant cancer cells.

### 2.2. Photodissociation of Bidentate Ligands

**Figure 5:**
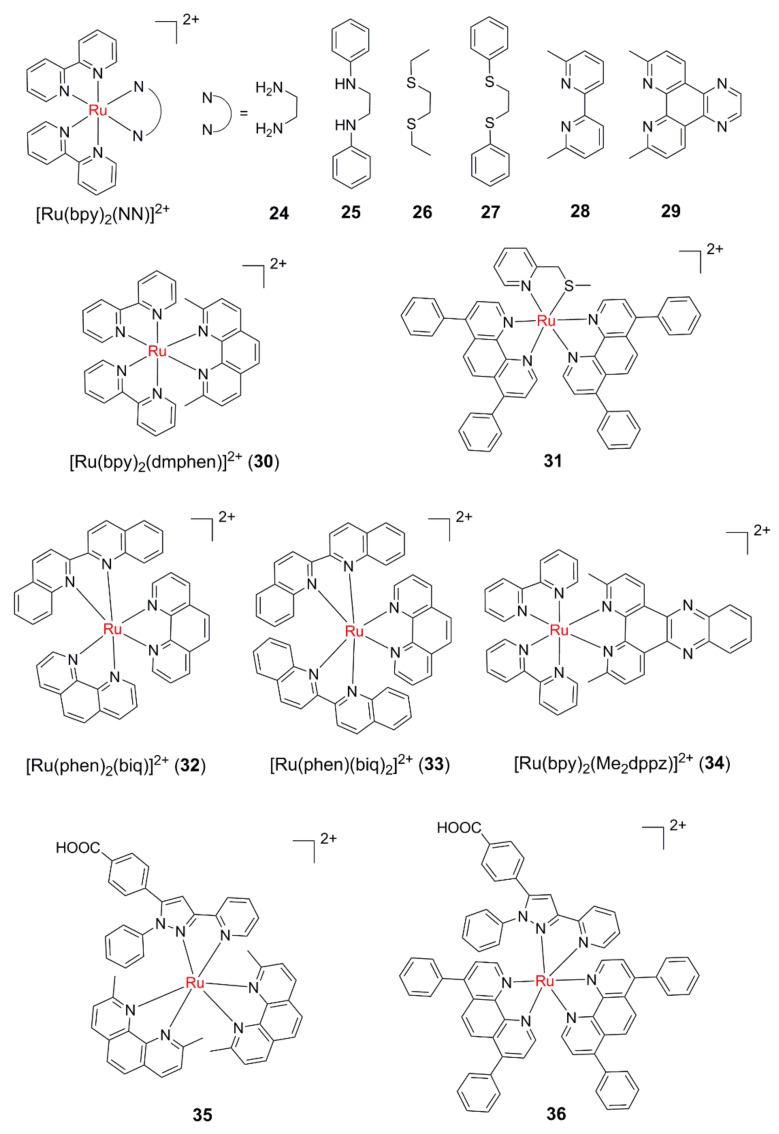
Ru(II) polypyridyl complexes with photolabile bidentate ligands.

Compared with monodentate ligands, the photodissociation of bidentate ligands is much more difficult due to the chelation effect. Although the two NH_3_ ligands of *cis*-[Ru(bpy)_2_(NH_3_)_2_]^2+^(**1**) can be readily photoreleased, the quantum yield of ethanediamine (en) ligand exchange with Cl^−^ for [Ru(bpy)_2_(en)]^2+^ (**24**, Figure 5) is only 0.002 [62]. Similar to **24,** the quantum yield of ligand exchange for **25** is only 0.003.

By taking some effective strategies, bidentate ligands can also serve as photolabile ones to expose two active sites at the same time. An effective strategy to improve the dissociation efficiency of bidentate ligands is to weaken the coordination ability of the coordination atoms. For example, compared with **24** and **25**, the photodissociation quantum efficiency of weaker bis-thioether ligands in **26** and **27** increase significantly, which are 0.019 and 0.016, respectively [62]. These two complexes can effectively reduce the mobility of linearized pUC18 plasmid DNA in electrophoresis upon irradiation, indicating that they can covalently bind to DNA and show potential in PACT.

Another effective strategy to improve the dissociation efficiency of bidentate ligands is to utilize steric bulk. Glazer and co-workers designed two new complexes **28** and **29** [63]. Through the substitution of two methyl groups on the 6 and 6′ positions of bpy, and **2** and **9** positions of dpq (dpq = dipyrido [3,2-*f*:2′,3′-*h*]-quinoxaline), steric bulk was successfully introduced into **28** and **29**. When the two complexes are illuminated, Me_2_bpy and Me_2_dpq can be effectively dissociated, and the resulting hydrate can covalently bind to plasmid DNA, reducing the DNA mobility on agarose gels electrophoresis. Interestingly, **29** can photocleave and photobind DNA at the same time, which indicates that photosensitization of singlet oxygen can compete with the ligand dissociation decay pathway. It was worth mentioning that the photo-cytotoxicity increased by **2** orders of magnitude for **28** and **29**. According to the same idea, a series of Ru(II) complexes with steric bulk have been studied in depth [64,65,66,67,68].

Khnayzer et al., reported a sterically crowned complex **30**, which can release either bpy or dmphen (2,9-dimethyl-1,10-phenanthroline) ligands upon visible light irradiation in water [65]. It is noteworthy that the phototoxicity of **30** towards the ML-2 Acute Myeloid Leukemia cell line appears to come from dmphen rather than the bis-aqua photoproduct [Ru(bpy)_2_(H_2_O)_2_]^2+^. In another study, by comparing the ligand toxicity, lipophilicity, and cellular uptake of complexes **28** and **31**, Bonnet et al. found that the phototoxicity of **28** comes from the photoreleased dmbpy ligand, whereas the only phototoxicity species is the bis-aqua photoproduct in **31** [69]. A series of Ru polypyridyl complexes based on the nontoxic 3-(methylthio)propylamine ligand were reported. It was found that by fine-tuning the lipophilicity and steric strain, the Ru-based PACT prodrugs can be phototoxic [70].

2,2′-biquinoline ligand (biq) not only has steric bulk but also extends the absorption wavelength of the Ru(II) complexes due to its conjugated structure. In accordance with this expectation, the MLCT absorption maximums of complexes **32** and **33** were extended to 525 nm and 550 nm, respectively [66]. Complexes **32** and **33** could release the biq ligand, which resulted in the retarded electrophoresis rate of plasmid DNA and phototoxicity towards HL60 cells under red light irradiation. In addition, Glazer’s group used cross-linked polymeric assemblies to load the strained Ru(II) complexes for the treatment of cancer and found that using polymeric nanoassemblies is a promising method to improve the pharmacological properties of Ru(II) complexes [67].

The Ruthenium complex [Ru(bpy)_2_(dppz)]^2+^ (dppz = dipyridylphenazine) displays the property of “DNA light switch”; that is, the emission of [Ru(bpy)_2_(dppz)]^2+^ is quite low in aqueous solution, and can be switched on in the presence of DNA. The dppz ligand can intercalate into DNA base pairs; thus, the hydrogen bond between two N atoms in the phenazine ring and water molecule is inhibited [71]. Although the Me_2_dppz ligand can cause steric clashes, the ligand photodissociation efficiency of [Ru(bpy)_2_(Me_2_dppz)]^2+^ (**34)** is very low. Meanwhile, the photodissociation process of the Me_2_dppz ligand is activated after DNA insertion, resulting in covalent binding of the complex to DNA after ligand photodissociation [72]. Further studies revealed that the ligand photodissociation of **34** is through the so-called “associative” mechanism; that is, the complex first coordinates with the exchange ligand to form seven coordination intermediates and then loses the coordination hindrance ligand Me_2_dppz [73].

Recently, to expand the applications of sterically congested Ru(II) polypyridyl complexes in PACT, Glazer al et. investigated the photochemical and biological properties of a series of Ru(II) complexes with photolabile pyridyl-pyrazol(in)e ligands [74]. Due to the introduction of a carboxylic, complexes **35** and **36** exhibited the largest PI values of 146 and 59, respectively, by a remarkable reduction of dark cytotoxicity.

## 3. Extending the Photoactivation Wavelength

Similar to other phototherapies, the wavelength of photoactivation for PACT agents is important for clinical applications. The ideal phototherapy window is between 650 and 850 nm [75], while photoactivating most Ru(II) complexes mentioned above requires high-energy visible light (< 500 nm), which may be harmful to normal tissues [76] and has poor tissue penetration [77]. How to extend the photoactivation wavelength to the ideal phototherapy window has become one of the important problems to be solved.

**Figure 6:**
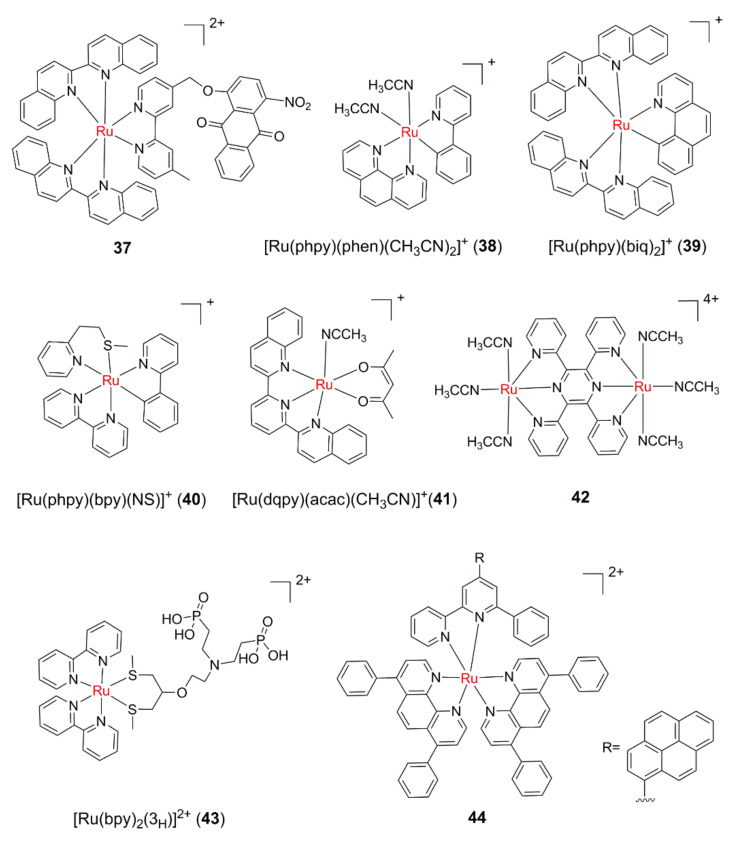
Photoactivatable Ru(II) polypyridyl complexes upon long-wavelength light irradiation.

As mentioned above, the large conjugated structure of the biq ligand obviously extends the photoactivation wavelengths of complexes **32** and **33**. On the one hand, the large conjugate system reduces the π* orbital energy of the biq ligand, thus reducing the energy of ^1^MLCT [t_2g_(Ru) to π*(biq)]. On the other hand, it causes steric hindrance, which reduces the ^3^MC energy through the distortion of the coordination field, and consequently ensures that efficient ligand dissociation can still occur upon low photon energy excitation. Recently, a nitro-anthraquinone group was attached to a biq-ligand-based Ru(II) complex by Wang and colleagues, endowing the resultant complex **37** (Figure 6) with multiple anticancer mechanisms upon irradiation in the phototherapy window [78]. It was found that **37** can release a biq ligand upon 600 nm irradiation, along with generating O_2_^•−^ and oxidizing NADH/NADPH (β-nicotinamide adenine dinucleotide phosphate).

When the ring-metalized-ligands (such as 2-phenylpyridine, phpy) coordinate with Ru, the strong electron-donating capacity of carbon anions can significantly increase the t_2g_ orbital energy of Ru(II), thus greatly prolongs the MLCT absorption wavelength. Using this strategy, Turro’s group designed and synthesized the complex *cis*-[Ru(phpy)(phen)(CH_3_CN)_2_]^+^ (**38**) [79]. The MLCT absorption peak of Ru(II) to phen ligand was at 490 nm, and the tail band extended to the phototherapy window. The quantum efficiency of ligand exchange between CH_3_CN and Cl^-^ was up to 0.25 upon 450 nm excitation. Different from the wavelength dependence of photoinduced ligand dissociation mentioned above, under 400 nm light irradiation, the ligand exchange efficiency of **38** decreased to 0.08. It is believed that the MLCT transition at 400 nm is mainly from Ru(II) to phpy ligand. Since the absorption band at 400 nm also contains too much of the ππ* transition component of the phpy ligand, the ligand photodissociation efficiency decreases instead of increasing. Turro’s group further designed and synthesized the complex [Ru(biq)_2_(phpy)]^+^ (**39**) by combining the phpy and biq ligands [45]. Cyclometallation results in a redshift of the MLCT absorption maximum of **39** by about 100 nm relative to that of **32** and **33.** Although **39** exhibited a distorted octahedral geometry, photoinduced ligand exchange did not occur. DFT calculations indicated that the difference of reactivity in **39** was ascribed to increased energy of ^3^MC states resulting from the bonding of the strong σ-donor phpy ligand. Bonnet et al. investigated the cyclometalated Ru complex **40**, which contained photolabile N,S bidentate ligand and found that **40** can be active under 521 nm irradiation in CH_3_CN, which makes it the first cyclometalated Ru complex capable of undergoing photosubstitution of a bidentate ligand [80].

Turro et al. introduced new ancillary ligand platforms that consist of anionic acetylacetonate-based ligand along with tridentate 2,6-di(quinolin-2-yl)pyridine (dqpy) ligand into complex **41** [81]. Acetylacetonate ligands are strong π-donor ligands that used to destabilize the HOMO (highest occupied molecular orbital), whereas the dqpy ligand with its extended conjugation lowers the LUMO (lowest unoccupied molecular orbital) energy; both effects make the ligand substitution of **41** accessible with near-infrared (NIR) light (≥ 715 nm).

The MLCT absorption band of binuclear or polynuclear Ru(II) complexes constructed with bridged ligands tends to be significantly redshifted compared with that of corresponding mononuclear complexes [82,83,84]. As a result, the bridging concept was utilized to design the first dinuclear Ru(II) complex (**42**) capable of undergoing ligand dissociation at both metal centers upon ≥ 610 nm irradiation in H_2_O [85]. To further explore the effect of the bridging ligand on Ru(II)-based PACT agents, Dunbar et al. used quinoxaline-functionalized bridging ligand platforms to prepare corresponding binuclear complexes [86]. These complexes were capable of absorbing green light with tails extending beyond 650 nm, which is a promising feature for applications in PACT.

In recent years, upconversion nanoparticles (UCNPs), which produce high-energy light under NIR excitation, were successfully used to extend the photoactivation wavelength of Ru-based PACT drugs [87,88,89,90,91]. More recently, Bonnet et al. reported the synthesis and photochemistry of the Ru(II) polypyridyl complex [Ru(bpy)_2_(3_H_)]^2^^+^ **(43)** [92], where 3_H_ is a photolabile bis(thioether) ligand. Complex **43** was bound to the surface of Lanthanoid-doped UCNPs through its bis(phosphonate) group, thereby creating an H_2_O-dispersible, thermally stable nanoconjugate. The incorporation of the neodymium-doped shell allowed for activation of **43** by 796 nm irradiation, which prevents the undesired heating seen with conventional UCNPs activated at 980 nm.

Additionally, two-photon absorption has been utilized for photoactivation of Ru(II) complexes, in which the prodrugs are activated by radiation in the PDT window [93,94,95,96]. Recently, by simply using a pyrene-modified-terpyridine (tpy-Py) as the labile bidentate ligand, Zhou et al. designed and synthesized a promising Ru(II) polypyridine complex **44**, which shows dual PACT and PDT activity upon efficient two-photon excitation in the NIR region [97]. Complex **44** can potently inactivate hypoxic and cisplatin-resistant cancer cells upon two-photon excitation at 800 nm.

## 4. Ru(II) PACT Agents with Multiple Functions

As previously described, photoinduced ligand dissociation of the Ru(II) polypyridine complex was achieved by the thermal population of ^3^MC states from the lower-lying ^3^MLCT states. Therefore, the rational and precise regulation of the energy levels of MLCT, MC, and LC states can theoretically regulate the ratio of each decay pathway, which may endue the complexes with multiple functions.

### 4.1. Ru(II) Complexes with Dual PACT and PDT Activity

**Figure 7:**
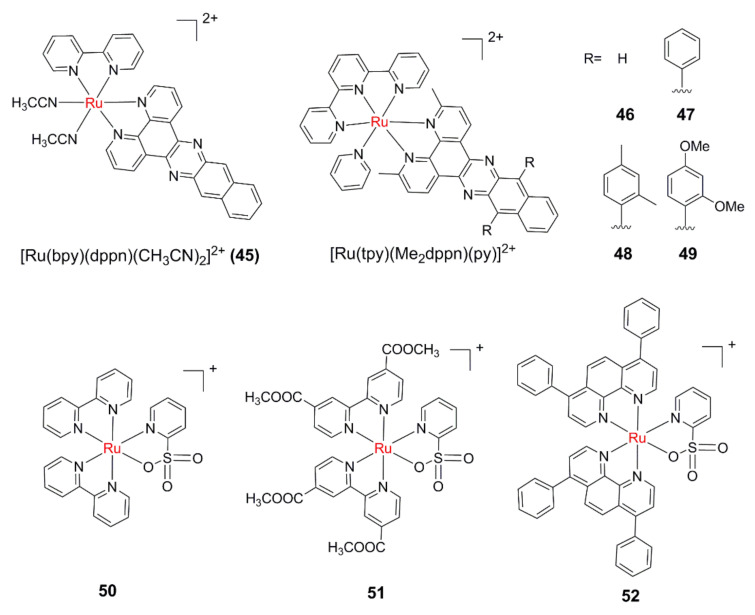
Ru(II) complexes with dual PACT and PDT activity.

Based on the classical photoinduced ligand dissociation of complex **3**, Dunbar’s and Turro’s groups further introduced a dppn (benzo[i]dipyrido-[3,2-a;2′,3′-c]phenazine) ligand with low ^3^ππ* energy level [98]. The complex [Ru (bpy)(dppn)(CH_3_CN)_2_]^2+^ (**45**, Figure 7) can not only dissociate the CH_3_CN ligands through ^3^MC state to form hydrate [Ru(bpy)(dppn)(H_2_O)_2_]^2+^ under 460 nm illumination (Φ < 1%), but can also produce ^1^O_2_ (Φ = 0.72, in methanol) thanks to the long-lived ^3^ππ* (dppn) state, realizing the combination of PACT and PDT in one Ru(II) complex. Although the ligand photodissociation efficiency of **45** was relatively low, complex **45** was highly cytotoxic towards HL60 cancer cells under 466 nm irradiation, with a PI value of 1110, which is much higher than that of the model complexes [Ru(bpy)_2_(dppn)]^2+^ (PI = 282) and **3** (PI = 6.4). By utilizing the steric bulk, Turro’s group further expanded the study of these Ru(II) complexes with dual photoreactivity [99,100,101,102], such as complex [Ru(tpy)(Me_2_dppn)py]^2+^ (**46**, tpy = terpiridine).

Recently, a series of Ru(II) complexes **47**–**49** containing arylated derivatives of the ligand Me_2_dppn were reported [103]. Complexes **47**–**49** displayed greater lipophilicity relative to **46**, and can interact with DNA in an electrostatic manner instead of intercalation. Quantum yields for photodissociation of the monodentate pyridines in **47**–**49** were about three times higher than that of **46**, whereas quantum yields for ^1^O_2_ generation were about 10% lower. Complexes **47**–**49** showed photoactivated toxicity in breast and prostate cancer cell lines, and PIs were lower than that of **46**.

The complexes **50** and **51** were designed and synthesized by using pyridine sulfonic acid (py-SO_3_) as the leaving group [104,105]. It was found that **50** and **51** can undergo py-SO_3_ dissociation upon visible light irradiation and produce reactive free radical species, and is therefore able to photobind and photocleave DNA simultaneously in hypoxic conditions. Unexpectedly, poor cell phototoxicity was observed for these two complexes. Lately, another py-SO_3_- based complex **52** was synthesized and studied by the same research group [106], which displayed efficient phototoxicity towards a series of cancer cells, including cisplatin-resistant human ovarian adenocarcinoma cells (SKOV3) and human lung adenocarcinoma cells (A549). Detailed studies indicated that the high cytotoxicity of **52** may be attributed to its enhanced cell uptake and nuclear accumulation levels. Patra et al. designed and synthesized two Ru complexes of saccharin with dipyridoquinoxaline and dipyridophenazine. Upon irradiation with UV-A light of 365 nm, both complexes can undergo photoinduced dissociation of saccharin ligand and generate reactive oxygen species, showing dual PDT and PACT activities [107].

### 4.2. The Combination of Ru(II)-Based PACT and Bioactive Ligands

By replacing the photolabile ligands in Ru-based PACT drugs with bioactive molecules, such as small molecule drugs and enzyme inhibitors, the resulting Ru(II) complexes may possess dual activity. Upon light activation, these complexes can release active ligands that may directly kill cancer cells, while the Ru(II) aqua species are able to damage DNA simultaneously.

**Figure 8:**
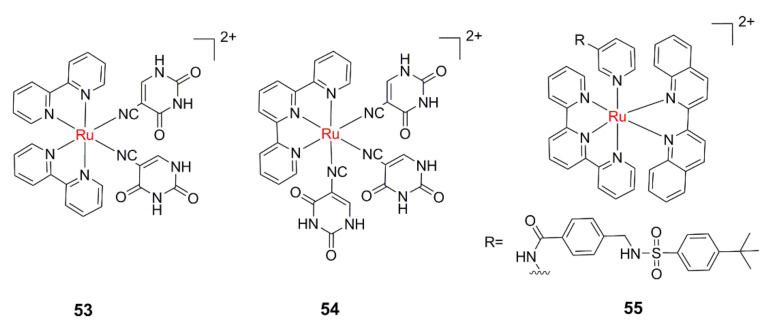
Ru(II) PACT agents with photolabile bioactive molecules.

Dunbar’s and Turro’s groups designed and synthesized **53** and **54** (Figure 8) using 5-cyano uracil (5CNU), a derivative of the chemotherapeutic drug 5-fluorouracil (5FU), as the leaving ligand [108,109]. The results of the cytotoxicity experiment showed that **54** showed similar cytotoxicity to the free 5CNU ligand at the same concentration under 400 nm illumination. Bonnet et al. reported a water-soluble Ru(II) complexes **55**, which can photorelease a cytotoxic nicotinamide phosphoribosyltransferase (NAMPT) inhibitor in an oxygen-independent manner [110]. A 3–4-fold increase in cytotoxicity was found upon red-light irradiation for **55**, whether the cells were cultured with 1% or 21% O_2_.

Until now, the anticancer agent CHS-828 [111], the imidazole-based cytotoxic drug econazole [112], the anti-tuberculosis drug isoniazid [113], the inhibitor of cathepsin K [114], cytochromes P450 [115] and CYP17A1 [116], etc. successfully photoreleased from Ru-based PACT agents were reported.

Due to the dual PACT and PDT activity presented by **46**, [Ru(tpy)(Me_2_dppn)] fragment was used to cage bioactive molecules for the purpose of achieving triple functions. Epoxysuccinyl-based inhibitors of cathepsin B (CTSB), a cysteine protease strongly associated with invasive and metastatic behavior, was conjugated to [Ru(tpy)(Me_2_dppn)] fragment by Kodanko et al. The study confirmed that the conjugate was capable of releasing ligand to form Ru(II) active center, generating ^1^O_2_ under light conditions, and irreversibly inhibiting CTSB, eventually causing efficient cell death [102].

### 4.3. Dual-Activatable Ru(II) PACT Agents

Although the photoactivation process has been effective in increasing drug selectivity, unnecessary light irradiation, such as sunlight, may still activate the prodrugs in normal tissues (such as skin), resulting in side effects. The introduction of other tumor-microenvironment-related activation factors, such as high-level GSH (glutathione), low pH values, etc., may improve the selectivity of Ru(II) PACT agents further.

**Figure 9:**
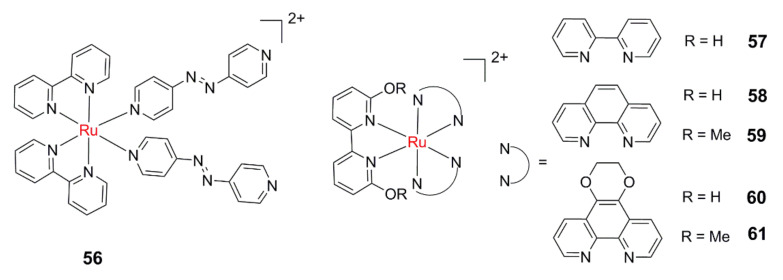
Dual-activatable Ru(II) PACT agents.

Wang et al. designed and synthesized a potential GSH—responsive Ru(II) PACT agent [Ru(bpy)_2_(py-N = N-py)_2_]^2+^ (py-N = N-py = 4,4′-azopyridine, **56**, Figure 9) by tethering a redox-active azo group on the leaving ligand [117]. The azo group can effectively quench the ^3^MLCT state and prevent the population of the ^3^MC state, which makes the target complex very stable under illumination. The DNA covalent binding capability of **56** can only be activated after GSH reduction and visible light irradiation. The concentration of GSH in tumor tissue is usually several times higher than that in normal tissue, which is also considered as a potential target of tumor tissue. This kind of GSH responsive PACT agent is expected to have a higher selectivity for tumor cells.

Considering the inherent acidity surrounding cancer cells, Papish and co-workers reported a series of pH-activated PACT agents, which can be activated by light- and pH-triggered ligand dissociation [118,119,120]. At a low pH value (pH = 5), complexes **57**–**61** existed in their acidic form, and the quantum yields for photodissociation were higher than in deprotonated form. Further studies validated that these complexes can produce ^1^O_2_ under illumination. Thus, they investigated how synthetic changes to ligands and ligand protonation states can influence the quantum yields for ^1^O_2_ and photodissociation. Cytotoxicity studies showed that ^1^O_2_ formation is a more plausible cause of photocytotoxicity [120].

Recently, a CO/light dual-activatable Ru(II)-oligo-(thiophene ethynylene) (Ru-OTE) agent for lysosome-targeted multimodal cancer therapeutics was reported [121]. Upon the dual-triggering of CO and light, Ru-OTE can undergo ligand substitution and generate ^1^O_2_. Importantly, Ru-OTE can be directly photoactivated using a two-photon laser (800 nm) and inhibit tumor growth in a breast tumor model.

## 5. Ruthenium(II) Arene Complexes

The above-mentioned Ru-based PACT agents are all Ru(II) polypyridyl complexes; in fact, another large family of Ru(II) complexes, i.e., Ru(II) arene complexes, show attractive prospects in chemotherapy [122,123]. They have the general formula [(η^6^-arene)Ru(L)(X)]^n+^ (where L is a bidentate ligand and X is a leaving ligand (usually halogen), and exhibit a spatial “piano stool” structure. The antitumor activity of these complexes is usually attributed to the formation of hydrates following the dissociation of the X ligand. When X is a halogen, the dissociation of the X ligand is spontaneous and uncontrollable. By replacing the X ligand with another monodentate ligand, such as pyridine and its derivatives, and optimizing the bidentate ligand and aromatic ring, Ru(II)-arene-based PACT agents can also be obtained.

**Figure 10:**
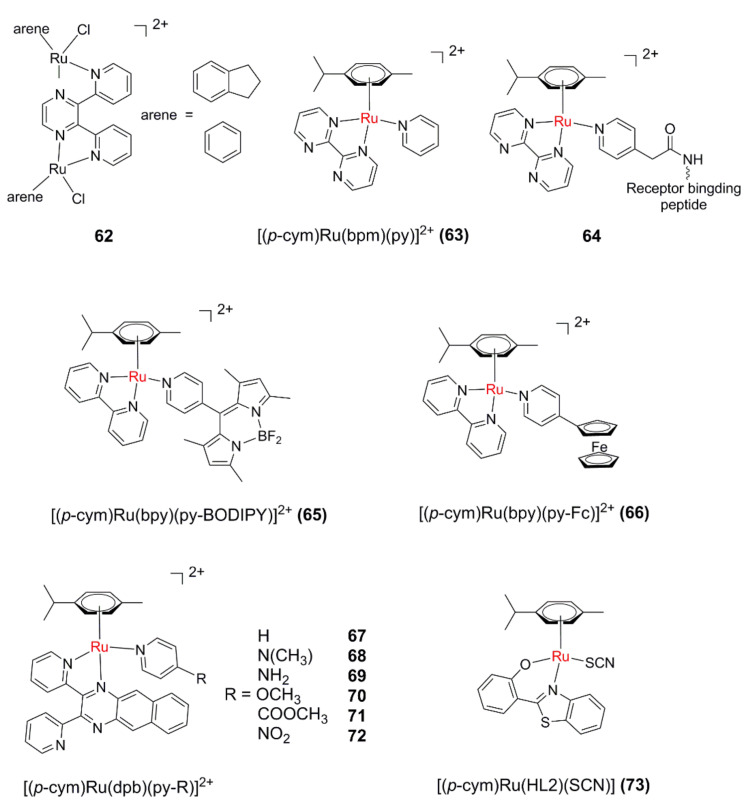
Ru(II)-arene-complexes-based PACT agents.

Sadler’s group reported a series of ruthenium arene binuclear complexes (**62**, Figure 10) in 2007 [124]. The binuclear complexes with indene or benzene as aromatic ligands can dissociate aromatic ligands under UVA light, and the resultant hydrate can form cross-linking with DNA.

Sadler’s group [125] further designed and synthesized [(*p-cym*)Ru(bpm)(py)]^2+^ (**63**) by introducing an electron-deficient bidentate ligand bpm (2,2′-bipyrimidine). When excited with visible light (400–600 nm), **63** can selectively dissociate the monodentate pyridine ligand and form a reactive aqua derivative able to bind to a DNA base. After that, they investigated the structure–photoactivity relationship of this kind of complex [126]. It seems the presence of a stronger electron-donating arene can promote the formation of the aqua adduct. In order to increase the tumor-targeting, a tumor-targeting factor was introduced into **63** to form **64** [127]. Upon irradiation with visible light, the monodentate ligand was dissociated, and the hydrate generated can covalently bind to guanine in the DNA sequence. Surprisingly, in the presence of oligomeric DNA, **64** can undergo photodissociation of aromatic ligands, and the resulting polyhydrate can covalently bind with two guanines at the same time.

Wang et al. designed [(*p-cym*)Ru(bpy)(py-BODIPY)]^2+^ (**65**) by modifying the BODIPY (4,4-difluoro-4-bora-3a,4a-diaza-s-indacene dye) group on the py ligand [128]. The introduction of the BODIPY group effectively extends the absorption wavelength of the complex to >500 nm. Interestingly, upon selective irradiation of the absorption band of the py-BODIPY ligand, the monodentate ligand can dissociate rapidly, and the resultant hydrate can covalently bind with the DNA base. It was found that photoinduced electron transfer from the BODIPY to the Ru(II) arene moiety plays an important role in the ligand dissociation. Based on the above results, they further synthesized [(*p-cym*)Ru (bpy)(py-Fc)]^2+^ (**66**) by introducing the ferrocene (Fc) group with redox activity on the py ligand [129]. The introduction of the ferrocene group makes **66** have a long-wavelength absorption band extending to 600 nm. Moreover, **66** can generate both hydroxyl radicals (·OH) and ^1^O_2_ along with photoinduced monodentate ligand dissociation, leading to phototoxicity against SKOV3 and A549.

An intriguing mononuclear Ru(II) arene complex [(*p-cym*)Ru(dpb)(py)]^2+^ (**67**) [130] with a dpb (2,3-bis(2-pyridyl)-benzoquinoxaline) ligand was synthesized and scrutinized by the same research group. The bulky features of the dpb ligand result in a strained coordination sphere of **67**, which leads to the photodissociation of both monodentate and bidentate ligands. The highly delocalized nature of dpb provides **67** with long-wavelength-absorbing properties and the ability to generate ^1^O_2_. The cytotoxicity test showed that the complex could effectively kill A549 cells under light conditions with a PI value of 6.9. Subsequently, a series of [(*p-cym*)Ru(dpb)(py-R)]^2+^complexes (**68**–**72**) were synthesized and compared to disclose the substituent effect on the py ligand [131]. It seems that the electron-withdrawing groups facilitate both ^1^O_2_ generation and ligand photodissociation.

García et al. investigated a neutral Ru(II) arene complex **73** bearing a benzothiazole ligand (HL2) as the bidentate ligand [132]. The results of this study indicated that the arene ligand is released after irradiation with UV light even in the absence of oxygen and leads to cytotoxicity enhancement, thus revealing the potential of **73** as a prodrug for PACT.

## 6. Conclusions and Future Perspectives

The research on Ru(II)-based PACT agents has been a hotspot in the field of metal anticancer drugs in recent years. Thanks to the efforts of many groups, continuous progress has been achieved in recent years. However, there is still a long way to realize the clinical applications of these Ru(II)-based PACT agents.

First, the photoactivation wavelength needs to be further extended. However, prolonging the MLCT absorption wavelength will increase the energy gap between the ^3^MLCT state and the ^3^MC state, which will, unfortunately, reduce the ligand dissociation efficiency. How to prolong the activation wavelength without sacrificing the ligand dissociation efficiency is full of challenges.

Second, most of the current studies focus on the ligand photodissociation-related photophysical and photochemical properties, while less attention is paid to the structure–activity relationship of antitumor activity. Many Ru(II) complexes displayed efficient photoinduced ligand dissociation; however, little anticancer activity was observed. Therefore, in addition to the ligand photodissociation, more efforts are needed to disclose how the other factors (such as DNA binding, cellular uptake, subcellular localization, etc.) influence the anticancer activity of Ru(II) PACT agents.

Third, the real cellular anticancer targets of Ru(II) PACT agents are still unknown. DNA is proposed as the potential target, and indeed many studies have verified that Ru(II) complexes with photolabile ligands can form Ru–DNA covalent binding in solutions after photoinduced ligand dissociation. However, some of the reported Ru(II) complexes with efficient PACT activity cannot attend the nucleus, which may hint to us that DNA should not be the only target. To disclose the real anticancer mechanism can undoubtedly promote the rational design of efficient Ru(II) PACT agents.

Fourth, among the existing reports on Ru(II)-based PACT agents, only a few studies were conducted at the living animal level. Although some promising results in vitro have been achieved so far, the lack of research in vivo is not conducive to promoting the clinical application of these complexes. Obviously, Ru(II) PACT agents may not be suitable to treat all kinds of cancers; thus, finding the possible indications of Ru(II) PACT is meaningful.

The research of Ru(II)-based PACT agents may still be at the early stage. We hope the current review can inspire more researchers with different backgrounds to enter this interesting field.

## Data Availability

Not applicable.
